# Revealing local structural properties of an atomically thin MoSe_2_ surface using optical microscopy

**DOI:** 10.3762/bjnano.13.49

**Published:** 2022-07-01

**Authors:** Lin Pan, Peng Miao, Anke Horneber, Alfred J Meixner, Pierre-Michel Adam, Dai Zhang

**Affiliations:** 1 Institute of Physical and Theoretical Chemistry, Eberhard Karls University of Tübingen, Auf der Morgenstelle 15, 72076 Tübingen, Germany; 2 Laboratoire Lumière, nanomatériaux et nanotechnologies – L2n, Université de Technologie de Troyes & CNRS EMR 7004, 12 Rue Marie Curie, CS42060, 10004 Troyes Cedex, France; 3 Center for Light-Matter-Interaction, Sensors and Analytics (LISA+), Eberhard Karls University of Tübingen, Auf der Morgenstelle 15, 72076 Tübingen, Germany

**Keywords:** copper phthalocyanine, local structure, molybdenum diselenide, optical spectroscopy, surface-enhanced Raman spectroscopy

## Abstract

Using a triangular molybdenum diselenide (MoSe_2_) flake as surface-enhanced Raman spectroscopy (SERS) platform, we demonstrate the dependency of the Raman enhancement on laser beam polarization and local structure using copper phthalocyanine (CuPc) as probe. Second harmonic generation (SHG) and photoluminescence spectroscopy and microscopy are used to reveal the structural irregularities of the MoSe_2_ flake. The Raman enhancement in the focus of an azimuthally polarized beam, which possesses exclusively an in-plane electric field component is stronger than the enhancement by a focused radially polarized beam, where the out-of-plane electric field component dominates. This phenomenon indicates that the face-on oriented CuPc molecules strongly interact with the MoSe_2_ flake via charge transfer and dipole–dipole interaction. Furthermore, the Raman scattering maps on the irregular MoSe_2_ surface show a distinct correlation with the SHG and photoluminescence optical images, indicating the relationship between local structure and optical properties of the MoSe_2_ flake. These results contribute to understand the impacts of local structural properties on the Raman enhancement at the surface of the 2D transition-metal dichalcogenide.

## Introduction

Two-dimensional (2D) materials have garnered interest for the next generation of optoelectronic and electrochemical devices, mainly owing to their fascinating optical and electronic properties [1]. In particular, the optical absorption, direct bandgap, and broken inversion symmetry of 2D transition-metal dichalcogenide (2D-TMDC) monolayers make these materials promising candidates for light-emitting diodes, photodetectors, field-effect transistors, valleytronics, and nonlinear optics [2–8]. Many interesting phenomena can be observed, mainly due to the presence of structural irregularities such as point defects, edges, boundaries, and the formation of contaminants in the process of 2D-TMDC growth [9–13]. These structural effects strongly influence the optical and electronic properties of 2D-TMDC materials. Optical second harmonic generation (SHG) spectroscopy has been recently used to study the presence of mid-gap states in the electronic band structure of WS_2_ flakes, which are induced by sulfur vacancies [14]. In addition, point defect-induced trions in monolayer WS_2_ on a nonconducting substrate can be visualized via photoluminescence in order to precisely explore the exciton binding energy [15]. The optical properties of edges and grain boundaries in 2D-TMDC materials have also been characterized by photoluminescence spectroscopy. Owing to the larger population of charge carriers, the photoluminescence from these structural defects of monolayer WS_2_ originates from the biexcitons under high-power excitation [16]. Interestingly, tilt boundaries in monolayer MoS_2_ induce strong photoluminescence enhancement and slightly decrease the in-plane electrical conductivity, whereas mirror twin boundaries lead to photoluminescence quenching and increase the conductivity [17]. Tip-enhanced Raman spectroscopy has been successfully used to visualize the point defect-related Raman vibrational modes in monolayer WS_2_ and edge defect-induced band bending of the conduction band at K and Q states in few-layer MoS_2_ [9–10]. All in all, structural irregularities play a crucial role in the modification of the electron band structure in 2D-TMDCs, further ruling their optical and electronic properties. Therefore, the relationship between structural irregularities and properties of 2D-TMDC materials has been intensively explored recently.

Surface-enhanced Raman spectroscopy (SERS) has been used as an ultrasensitive and nondestructive spectroscopic technique for fundamental investigations of light–matter interactions down to the single-molecule detection level [18–19]. The Raman enhancement originates from an electromagnetic mechanism, provided by the excitation of surface plasmons, and a chemical mechanism which is related to the modification of Raman polarizability of molecules [20]. It has been reported that 2D materials, including graphene and 2D-TMDC materials, are unique platforms for SERS investigations based on the chemical mechanism [21]. Recently, enhanced Raman signals of rhodamine 6G (R6G) molecules on an oxygen plasma-treated MoS_2_ flake were reported, because the symmetry of the R6G molecule can be modified through the interaction with local dipoles in plasma-treated MoS_2_ [22]. Additionally, the electronic band structure of MoS_2_ can be significantly modified after oxygen incorporation into MoS_2_. The charge transfer from the valence band of partially oxidized MoS_2_ to the LUMO of R6G can be tuned in resonance with the excitation energy, leading to a giant chemical enhancement on partially oxidized MoS_2_ [23]. It has been also reported that the energy levels and orientation of the Raman-active probe molecule on graphene could strongly influence the Raman enhancement. Benefiting from the face-on molecular orientation and molecular energy levels in the vicinity of the Fermi level of graphene, the charge-transfer effect can become more pronounced [24–25]. In summary, the structural irregularities in 2D materials and molecular probe both can impact the strength of molecule–substrate interactions and then modify the Raman polarizability of the molecule; thus, it is essential to investigate the dependency of chemical enhancement on the local structure of 2D-TMDC materials.

In this article, the structure-related optical properties of a triangular MoSe_2_ flake covered with a 5 nm film of CuPc molecules are investigated using custom-built confocal optical microscopy assisted by a parabolic mirror. Both azimuthally and radially polarized doughnut beams are used for evaluating the polarization-dependent Raman enhancement at the MoSe_2_ surface. With the combination of SHG and photoluminescence microscopy, structural properties of MoSe_2_ flakes, such as the structural irregularities, thickness, and crystal stacking, are revealed. The dependencies of Raman and photoluminescence signals of the MoSe_2_ flake on polarization and local structure are compared and discussed. These results contribute to understand the impact of local structural properties on the Raman enhancement at 2D-TMDC monolayer surfaces.

## Results

**Figure 1:**
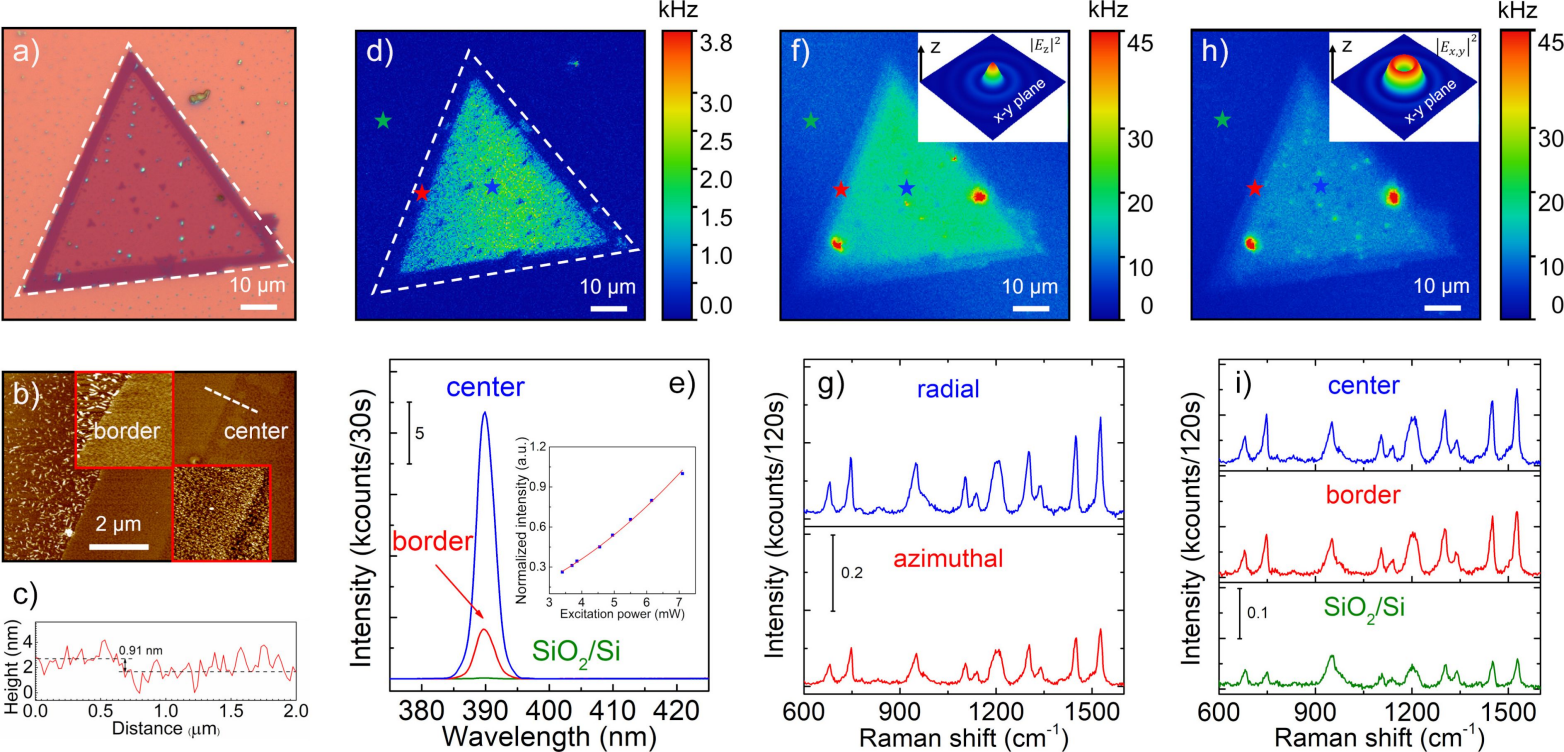
Optical properties of a triangular MoSe_2_ flake covered with a thin film of 5 nm of CuPc. (a) Bright-field optical image of CuPc/MoSe_2_. (b) AFM topographic image of CuPc/MoSe_2_. The upper and lower insets show high-resolution AFM images (2 × 2 μm) scanned from the SiO_2_/Si substrate to the border of MoSe_2_ flake and from the border to the center of MoSe_2_ flake, respectively. The dashed white line represents the position where the height profile was taken. (c) Height profile of CuPc/MoSe_2_. (d) SHG map of CuPc/MoSe_2_. The dashed white triangle denotes the real size of the triangular MoSe_2_ flake. SHG measurements were performed using a custom-built confocal microscope with a femtosecond pulsed laser (89.8 fs, 40 MHz, 780 nm, linear polarization). The excitation power used in the SHG map in (d) is 5.80 mW. (e) SHG spectra collected from CuPc/MoSe_2_. The inset shows the integrated SHG intensity as a function of the excitation power. (f) and (h) show optical images of CuPc/MoSe_2_ under the excitation of a radial and an azimuthally polarized laser beam, respectively. The optical measurements are performed using a custom-built confocal microscope with a 636 nm diode laser. Raman and photoluminescence spectra are collected simultaneously in the optical images. The blue, red, and green stars indicate the positions where the optical spectra are collected at the center of the MoSe_2_ flake, the border of the MoSe_2_ flake, and the SiO_2_/Si substrate, respectively. The insets in (f) and (h) show the diagrams of electric field intensity distribution in the *x*–*y* plane in the focus of a radially and an azimuthally polarized laser beam (636 nm, continuous wave), respectively. The excitation powers of radially and azimuthally polarized beam were 76.0 μW and 75.0 μW, respectively. (g) Raman spectra collected from the center position of the CuPc/MoSe_2_ flake under the excitation with a radially and an azimuthally polarized laser beam, respectively. (i) Raman spectra measured at the different positions of the CuPc/MoSe_2_ flake using azimuthal polarization.

In this work, triangular MoSe_2_ flakes were chemically synthesized on a precleaned Si substrate coated with a thermally grown layer of SiO_2_. To investigate the Raman enhancement effect on a MoSe_2_ flake, we choose CuPc as a Raman probe, because CuPc exhibits a large Raman scattering cross section and an extremely weak photoluminescence signal. A thin film of 5 nm of CuPc was deposited on the triangular MoSe_2_ flakes through thermal vapor deposition. Figure 1a shows a bright-field optical image of CuPc/MoSe_2_. From the optical contrast, one can estimate that the thickness of the more transparent areas of the MoSe_2_ flake is smaller than that of other regions. To visualize the CuPc molecule distribution on the MoSe_2_ flake, atomic force microscopy (AFM) was used, and the results are shown in Figure 1b. The insets in Figure 1b are high-resolution AFM images of CuPc/MoSe_2_. The upper inset exhibits a step from the SiO_2_/Si substrate to the border of the MoSe_2_ flake, and the lower inset shows a distinct transition from the border to the center of the MoSe_2_ flake. The MoSe_2_ flake is fully covered by CuPc molecule aggregations, while on the SiO_2_/Si substrate some CuPc molecules aggregated to a rod-like particle, which has been also reported in the literature [24]. The height profile marked by the dashed white line in Figure 1b is visualized in Figure 1c. It suggests that the center of the MoSe_2_ flake is slightly thinner than the border. As shown in Supporting Information File 1, Figure S1, the center of the MoSe_2_ flake shows a Raman out-of-plane mode at 240 cm^−1^ (*A*_1_*_g_*) and another in-plane mode at 287 cm^−1^ (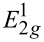), which is in good agreement with the Raman peaks of a MoSe_2_ monolayer [26]. Two Raman peaks at 241 cm^−1^ (*A*_1_*_g_*) and 285 cm^−1^ (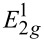) are observed at the border of the MoSe_2_ flake, which can be determined to be a MoSe_2_ bilayer [27].

SHG spectroscopy was performed to determine the crystal stacking structure of the MoSe_2_ flake. Figure 1d exhibits the SHG map of the flake shown in Figure 1a using a 780 nm femtosecond pulsed laser as the excitation source. The real size of the MoSe_2_ flake is indicated by the dashed white triangle. We find that the SHG signal is barely visible at the border of the MoSe_2_ flake compared to the center of the MoSe_2_ flake. Furthermore, the bright-field optical image reveals also some small triangular flakes on top of the underlying large flake, which appear dark in the SHG image in Figure 1d. The decreased SHG intensity at increasing layer thickness indicates a mirrored orientation of neighboring layers in the MoSe_2_ flake, which is typical for hexagonal 2H-phase MoSe_2_ [28–29]. Figure 1e shows the SHG spectra collected at the SiO_2_/Si substrate and the border and center of the MoSe_2_ flake. The exact positions are marked by the green, red, and blue stars in Figure 1d, respectively. The maximum of the spectra is located at 390 nm. Its second-order nonlinear property is verified by the excitation power-dependent measurement, and the SHG signal shows a quadratic power dependence, shown in the inset of Figure 1e.

The absorption spectrum of a CuPc film with a thickness of 10 nm on a glass substrate is shown in Supporting Information File 1, Figure S2. CuPc shows an intensive absorption region (called Q-band) from 500 to 800 nm. The absorption peaks situated at 627 and 696 nm are due to the second and the first π–π* transition of the phthalocyanine macrocycle, respectively [30]. Therefore, the Raman measurements for CuPc/MoSe_2_ are conducted using a 636 nm laser, which shows good resonance with the CuPc molecule. Figure 1f and Figure 1h illustrate the optical images of CuPc/MoSe_2_ under radially and azimuthally polarized beam excitation, respectively. Different from Figure 1d, the optical intensities in Figure 1f and Figure 1h are the sum of the Raman signals of CuPc molecule and the photoluminescence from the MoSe_2_ flake. It can be clearly seen that the optical signals from the CuPc/MoSe_2_ flake excited by a radially polarized beam show a stronger intensity than under azimuthally polarized beam excitation. Furthermore, the optical intensity at the border of the MoSe_2_ flake is weaker than that at the center. The insets in Figure 1f and Figure 1h show the calculated intensity distribution of the electric field in the *x*–*y* plane in the focus of the radially and azimuthally polarized laser beam, respectively. The center of a focused radial polarization beam exhibits mainly a *z*-direction field component in reference to the sample plane, and the transverse field in the focus is about 14 times weaker than the *z*-direction field component [31]. On the contrary, a focused azimuthal polarization beam has only a *x*–*y* plane field component (parallel to the sample plane) [32].

The Raman intensities of CuPc show a clear dependency on the excitation polarization and the local structural properties of the underlying MoSe_2_ flake. Figure 1g shows the Raman spectra collected from the center position of the CuPc/MoSe_2_ flake in Figure 1f and Figure 1h, which is marked by the blue star. The Raman signal obtained at the center of CuPc/MoSe_2_ under radially polarized beam excitation is stronger than that under azimuthally polarized beam excitation. Taking the 1527 cm^−1^ vibrational mode as an example, the Raman intensity obtained using the radial polarization is nearly 1.75 times higher than that from the azimuthal polarization. Figure 1i and Figure S3, Supporting Information File 1, show the Raman spectra from different regions of CuPc on the MoSe_2_ flake with azimuthally and radial polarized beam excitation, respectively. Excited by the azimuthal polarization, the Raman intensity at 1527 cm^−1^ from the CuPc/MoSe_2_ flake is about three times stronger than that from the SiO_2_/Si substrate. Furthermore, we also observe that the Raman intensity of CuPc at the flake center is slightly higher than that at the border for both azimuthal and radial polarizations.

**Figure 2:**
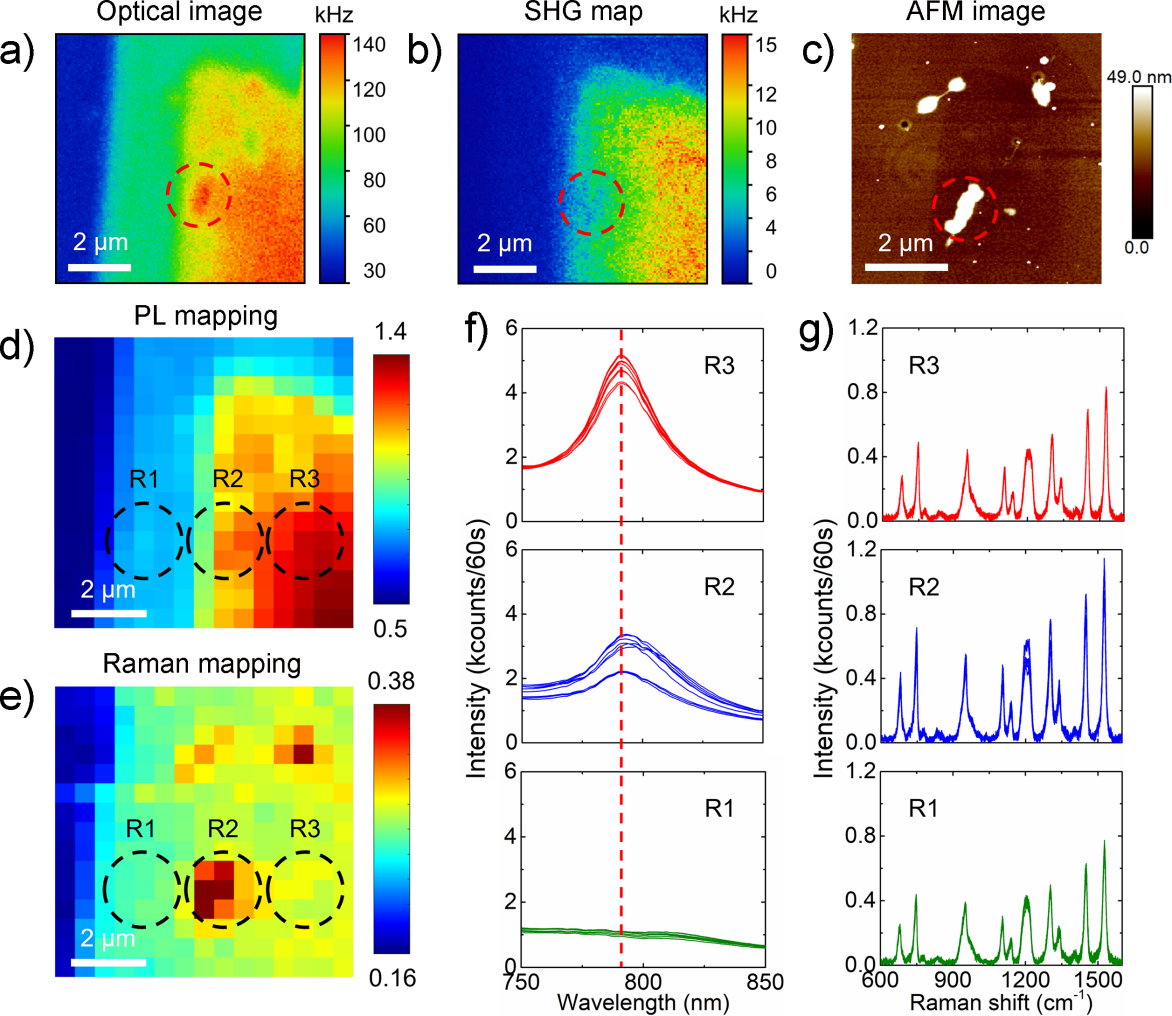
The local structure-related optical properties of CuPc/MoSe_2_. (a) Linear optical image of CuPc/MoSe_2_ with a scan area of 8 × 8 μm. Raman and photoluminescence signals are collected simultaneously in the linear optical images. (b) SHG map of the corresponding region of CuPc/MoSe_2_. (c) AFM topographic image of the corresponding region of CuPc/MoSe_2_. (d) Photoluminescence mapping image of the corresponding region of the photoluminescence peak from MoSe_2_ at 790 nm. (e) Raman mapping image of the corresponding region of CuPc Raman peak at 1527 cm^−1^. The color bars in (d) and (e) indicate the integrated optical intensity. The linear optical image and photoluminescence (Raman) mapping images are conducted using azimuthal polarization. (f) Photoluminescence spectra and (g) Raman spectra collected from three different regions (R1, R2, and R3) of the CuPc/MoSe_2_ flake.

The optical image and the SHG map with a scan area of 8 × 8 μm of CuPc/MoSe_2_ are shown in Figure 2a and Figure 2b, respectively. The optical intensities in Figure 2a are coming from the sum of CuPc Raman scattering and the MoSe_2_ flake photoluminescence. We find that the optical intensities in Figure 2a and the SHG intensity in Figure 2b show a pronounced correlation, which is exemplarily indicated by the dashed red circle in Figure 2a and Figure 2b. Figure 2c shows the AFM topographic image of the corresponding region of CuPc/MoSe_2_. The surface of the MoSe_2_ flake is covered by some nanoparticles marked by the dashed red circle, which were reported to be intermediate products (e.g., MoO*_x_*Se*_y_*) of the MoSe_2_ growth [33]. The by-products can be observed at the surface of both MoSe_2_ flake and SiO_2_/Si substrate and appear as small blue particles in Figure 1a. Figure 2d and Figure 2e show the hyperspectral images based on the photoluminescence and Raman intensities, respectively. The integrated photoluminescence intensities are plotted in the range of 750 to 850 nm, and the Raman intensities are the integrated peak area from the vibration mode of CuPc at 1527 cm^−1^. Three regions are marked in Figure 2d and Figure 2e as the border (R1), border to center (R2), and center (R3) regions of the MoSe_2_ flake, respectively. From R1 to R3, the photoluminescence mapping image shows a gradual increase of optical intensity. The optical spectra collected from these three regions are plotted in Figure 2f and Figure 2g. The photoluminescence peak is not visible for the region R1. From position R3 to R2, redshifts in the emission peak maxima by 3 to 6 nm can be observed, which depend on the location within the red circle in Figure 2e. The Raman signal at 1527 cm^−1^ from CuPc molecules is the highest in the R2 region, followed by R3 and R1. From Figure 2, one can see that SHG intensity, photoluminescence, and Raman enhancement are strongly related to the local structure of the MoSe_2_ flake.

## Discussion

**Figure 3:**
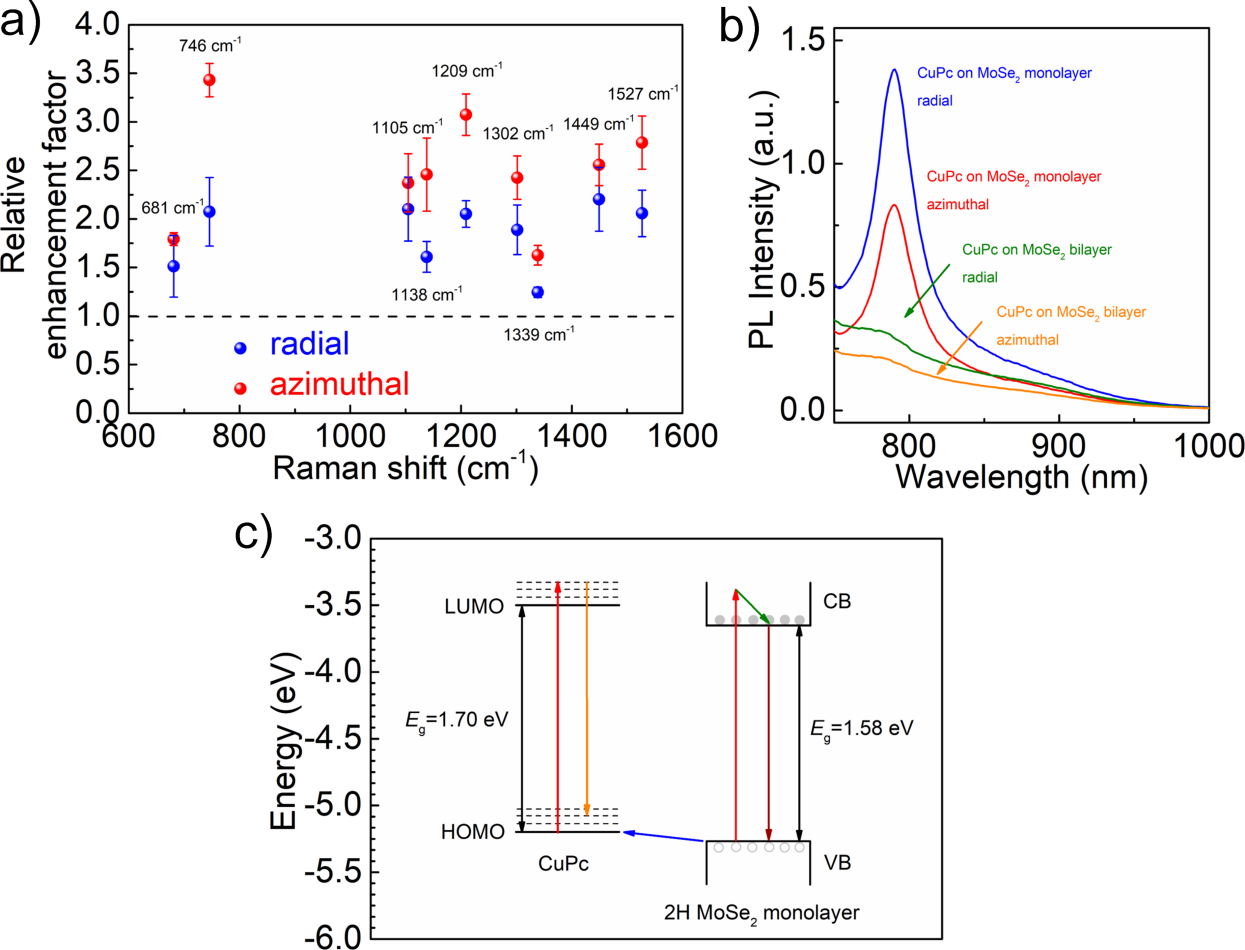
(a) Relative Raman enhancement factor at the center of the MoSe_2_ flake in reference to the SiO_2_/Si substrate. (b) Photoluminescence spectra collected at the border region (bilayer) and the center position (monolayer) of CuPc on MoSe_2_ flake. The photoluminescence peak is due to the direct bandgap emission in the MoSe_2_ monolayer. (c) A sketch of the energy level diagram at the interface between CuPc molecule and 2H MoSe_2_ monolayer. The ground-state charge transfer, represented by the blue arrow, occurs from the valence band of the MoSe_2_ monolayer to the HOMO of the CuPc molecule. Left side: the red arrow denotes the HOMO–LUMO transition in CuPc excited by a 636 nm laser. The yellow arrow indicates the Raman scattering process. Right side: the absorption process in the MoSe_2_ monolayer is indicated by the red arrow. The olive arrow denotes the non-radiative relaxation process. The photoluminescence emission in MoSe_2_ monolayer is represented by the wine red arrow.

A quantitative analysis of the enhancement factor of Raman modes of CuPc is shown in Figure 3a. Specifically, the vibrational modes located at 1339, 1449, and 1527 cm^−1^ are assigned to the C–C and N–C stretching vibrations of the isoindole ring [34–35]. The 746 cm^−1^ vibrational mode originates from the metal-bound N–M stretching vibration, and the 1138 cm^−1^ mode is attributed to the deformation of the isoindole ring system [36]. The Raman enhancement factor is calculated by dividing the Raman intensity of CuPc at the flake center by that on the SiO_2_/Si substrate. Interestingly, although excitation with radial polarization gives a stronger Raman intensity (e.g., 1527 cm^−1^) than excitation with azimuthal polarization, the enhancement factor of each Raman mode in Figure 3a with the azimuthal polarization (red) is higher. We also find that the Raman enhancement factors for different vibrational modes of CuPc are different, ranging from about 1.1 to 3.5. As reported in the literature [21], for monolayer CuPc on graphene, the vibrational modes at 1342, 1452, and 1531 cm^−1^ of CuPc are significantly enhanced, which is induced by the efficient charge transfer between molecule and substrate. Differently, for CuPc monolayers on a hexagonal boron nitride (h-BN) flake the vibrational modes at 749 and 1143 cm^−1^ show the largest Raman enhancement factor of all Raman peaks. This is attributed to the fact that these vibrational modes possess large dipoles leading to a strong dipole–dipole interaction with the underlying h-BN. The MoSe_2_ monolayer used in our work is a direct-bandgap semiconductor and has a polar covalent bond (Mo–Se). The Raman peaks of CuPc/MoSe_2_ at 1527, 1449, 1339, 1138, and 746 cm^−1^ are all enhanced compared to the case of CuPc on SiO_2_/Si, which indicates that both charge transfer and dipole–dipole interactions can occur.

Furthermore, these interactions appear to be more efficient under the excitation with the *E_x_*_,_*_y_* field component, which is parallel to the sample plane. To explain this phenomenon, we turn our discussions to the molecular orientation of CuPc on the MoSe_2_ flake. For a thin film thermally evaporated on a MoS_2_ flake surface, the CuPc molecule has been reported to adopt π-face-on orientation because the Cu metal center can link to a sulfur atom of MoS_2_ via axial coordination [37]. Considering the excitation polarization-dependent SERS enhancement in our experiments, we attribute the higher SERS enhancement factor with azimuthal polarization to the first-layer effect, where the charge transfer between the first layer of face-on lying CuPc and the MoSe_2_ flake is the most efficient. With increasing film thickness, we assume that the CuPc molecular orientation varies, such as adopting a tilt angle with respect to the sample surface. The evolution of molecular orientation was observed in a FePc thin film that was deposited on a MoS_2_ crystal surface. Using near-edge X-ray absorption (NEXAFS), it was observed a strong dichroism in FePc thin films thicker than 4.5 nm. The strongest intensity of the N 1s→π* orbital transition at grazing incidence implies that the molecules are predominantly flat-lying with respect to the substrate surface. At a film thickness of more than 4.5 nm, the molecules adopt small tilt angles and a high degree of ordering [38]. Here, due to the |*E_z_*|^2^ component in the focus of the radially polarized beam, the tilted CuPc molecules on the MoSe_2_ flake can still be excited, which would not be possible when using the pure |*E_x_*_,_*_y_*|^2^ component of a focused azimuthally polarized beam. To summarize, our results show that the CuPc molecules likely adopt a face-on orientation on the MoSe_2_ flake, similar to the case of MoS_2_ flakes. This molecular orientation facilitates the first-layer effect, which enables a high SERS enhancement factor when the molecules are excited with the |*E_x_*_,_*_y_*|^2^ component of the azimuthal polarization.

Regarding the photoluminescence spectra measured at the border (bilayer) and center (monolayer) regions of CuPc/MoSe_2_ with radial and azimuthal polarization (Figure 3b), we further discuss the charge-transfer direction between CuPc and MoSe_2_. The central wavelength of the photoluminescence peak is roughly at 790 nm, which is in good agreement with the exciton emission of monolayer MoSe_2_ [27]. The photoluminescence intensity of CuPc on bilayer MoSe_2_ is significantly lower than that of CuPc on monolayer MoSe_2_ for both radial and azimuthal polarizations, as shown in Figure 3b. Furthermore, the photoluminescence intensity of CuPc on monolayer MoSe_2_ integrated from 750 to 1000 nm under radial polarization is 1.63 times stronger than that under azimuthal polarization (Figure 3b), while the photoluminescence intensity of CuPc on bilayer MoSe_2_ excited by radial polarization is only 1.49 times higher compared to that with azimuthal polarization. Based on the above experimental results, the Raman enhancement mechanism at the interface between CuPc molecules and the 2H MoSe_2_ monolayer is illustrated in Figure 3c [27,36,39–41]. With an excitation of 636 nm, electrons in the HOMO of CuPc can be excited to the LUMO (red arrow, Figure 3c), leading to the formation of holes in the HOMO. As we discussed previously, with the azimuthal polarization excitation, the charge transfer from MoSe_2_ to CuPc is more efficient through the first-layer effect. In this case, more electrons in the valence band of MoSe_2_ can be involved in the Raman scattering process of CuPc through the ground-state charge transfer (blue arrow), which enhances the Raman scattering as shown by the yellow arrow [36]. In the meantime, less electrons can be excited from the valence band to the conduction band of the MoSe_2_ flake (red arrow). Thus, radiative electron–hole pair recombination (wine-red arrow) is less strong than it is with radial polarization, giving rise to the lower photoluminescence intensity shown in Figure 3c.

Raman scattering involves interaction between the incident light with the electrons in the ground state, the coupling between electrons in the excited state and phonons (vibrational modes), and scattered light radiation. Both charge transfer and dipole–dipole interaction can contribute actively to electron–phonon coupling [36]. With a face-on orientation of the CuPc molecules at the MoSe_2_ monolayer, the delocalized π electrons of CuPc overlap efficiently with the electron cloud of the MoSe_2_ monolayer. As the HOMO of CuPc is close to the valence band of the MoSe_2_ monolayer as shown in Figure 3c, it induces an increase of the electron density of states. According to Fermi’s golden rule, the electron transition probability rate *w_lk_* can be expressed as:


[1] $$w_{lk}=\frac{2\pi }{\hbar }g\left(E_{k}\right){\left|{H^{\prime }}_{kl}\right|}^{2},$$

where the *g*(*E_k_*) and 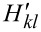 denote the density of states and the matrix element for the LUMO–HOMO transition, respectively. Therefore, the electron transition probability can be increased by increasing the density of states, which plays a significant role in the ground-state charge-transfer process, further leading to an enhancement of electron–phonon coupling and, consequently, an increase of the Raman scattering intensity [39].

Additionally, MoSe_2_ has a polar covalent bond (Mo–Se), which can induce interface dipole–dipole interactions with CuPc in a face-on orientation [24]. The dipole–dipole interaction will bring about a local symmetry-related perturbation, leading to an increase of the matrix element for the LUMO–HOMO transition (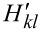) in Equation 1, which increases the electron transition probability rate (*w_lk_*) [21]. In particular, the vibration mode (746 cm^−1^) that is assigned to the metal-bound N–M stretching vibration shows a larger intrinsic dipole, leading to a dipole–dipole interaction with the Mo–Se bond of MoSe_2_. Thus, the Raman peak (746 cm^−1^) is more strongly enhanced under azimuthal polarization than under radial polarization, as shown in Figure 3a.

Furthermore, we show in Figure 2 that the SERS enhancement depends also sensitively on the local structural properties of the same flake under the same excitation polarization. The lowest Raman intensity from CuPc molecules is observed in the R1 region because of its bilayer property, which is verified by the vanishing photoluminescence intensity and its *A*_1_*_g_* Raman peak, which is shifted to higher wavenumbers. Regarding monolayer MoSe_2_, the SERS signal from the R2 region is higher than that from the R3 region. The lower photoluminescence intensity from the MoSe_2_ flake and the higher SERS signal observed at position R2 agrees very well with the ground-state charge-transfer mechanism explained in Figure 3c, which is shown to depend on the sample position. Furthermore, in the AFM image in Figure 2c, one can clearly see the presence of particle aggregates, which were reported to be oxidation products (MoO*_x_*Se*_y_* or MoO_3_) either through the CVD growth process or through aging in air. Sahoo et al. have reported that the aging of WSe_2_ flakes by exposure to air produces nanoparticles, which lead to a redshift by 2 to 4 nm in the photoluminescence peak position as compared to the pristine flake. They attributed the observed photoluminescence redshift to the formation of different states or strains in the presence of oxidation nanoparticles [42]. In Figure 2f, we see redshifts in the photoluminescence peak maxima, which are correlated to the high SERS intensity. Likely, new energy states through the presence of the oxidized nanoparticles were formed. The change in energy states is localized at the position of the particle aggregates, which is sensitively revealed by the SERS signal of CuPc. Therefore, our results clearly demonstrate that local flake structures can be revealed by monitoring the SERS enhancement at organic molecule/2D-TMDCs heterostructures.

## Conclusion

We demonstrated local structure-related optical properties of a CuPc/MoSe_2_ heterostructure using SHG, Raman, and photoluminescence spectroscopy and microscopy. The SHG intensity is significantly reduced when the thickness of the MoSe_2_ flake increases, which indicates that the crystal structure of the MoSe_2_ flake is the hexagonal 2H phase. The Raman enhancement of CuPc on MoSe_2_ obtained with azimuthal polarization is more pronounced than it is with radial polarization. This is mainly due to the first-layer effect, where ground-state charge transfer and interface dipole–dipole interactions between the first layer of face-on lying CuPc molecules and MoSe_2_ are more efficient. The SHG and photoluminescence properties of the MoSe_2_ flake are strongly influenced by structural irregularities, which are induced by intermediate products of MoSe_2_ growth. These local structure irregularities also affect the Raman enhancement of the MoSe_2_ flake.

## Experimental

### SHG measurements

SHG optical measurements are conducted by using a custom-built confocal microscope assisted with a parabolic mirror. The schematic diagram of the microscope is exhibited in Supporting Information File 1, Figure S4. A femtosecond pulsed laser (pro NIR_02508, Toptica Photonic) at an excitation wavelength of 780 nm (repetition frequency: 40 MHz, pulse duration: 89.8 fs) is used for the optical excitation. To reach a diffraction-limited focus, we use a parabolic mirror with a numerical aperture up to 0.998 in air for light focusing and emission signal collection. The SHG signals are selected using a 10 nm band pass filter and detected by micro-photon devices (MPD, MPD-PD-100-CTD) for optical imaging. The optical spectra are recorded using a spectrometer coupled with a thermoelectrically cooled charge-coupled device (CCD) camera (Acton SP 2500, Princeton Instruments).

### Raman and photoluminescence measurements

Raman and photoluminescence measurements are performed using a custom-built confocal microscope assisted with a parabolic mirror, as shown in Supporting Information File 1, Figure S4. In this microscope, either a 636 nm diode laser (PDL 800-D, Picoquant) or a 532 nm laser (NANO 250-532 max, QIOPTIQ) in continuous wave mode are coupled to the microscope. The linearly polarized laser beam is converted to either a radially or an azimuthally polarized beam by a mode converter formed by four quarters of half-wave plates. The details of the mode converter can be found in our previous publications [43–44]. The self-written program “PMCalc” by M. Sackrow, a modified program “Focused Fields” developed by M. A. Lieb and A. J. Meixner, is used to calculate the electric field intensity distribution in focus of radially and azimuthally polarized laser beams. The elastic scattering signals are filtered out by a configuration of two notch filters. The Raman and photoluminescence signals are collected by an avalanche photodiode (APD, SPCM-AQR-14, Perkin Elmer) for optical imaging. A spectrometer coupled with a liquid nitrogen-cooled CCD camera (Acton Research, SpectraPro 300i, Perkin Elmer) is used to obtain optical spectra.

### Preparation and characterization of CuPc molecules on MoSe_2_ flakes

The MoSe_2_ flakes were received from SixCarbon Technology (Shenzhen), synthesized on a SiO_2_/Si substrate using chemical vapor deposition. An ultra-thin film of CuPc with a thickness of 5 nm is deposited on the MoSe_2_ samples by vacuum thermal deposition. At a pressure of 10^−8^ mbar, CuPc powder (Sensient Imaging Technologies SA) is evaporated from a resistively heated crucible. The nominal deposition rate (0.2–0.3 nm/min) is monitored by a quartz crystal micro balance. A commercial optical microscope (MX50, Olympus) is used to obtain the bright-field optical images of CuPc/MoSe_2_. The topographic images of CuPc/MoSe_2_ are obtained with an atomic force microscope (Multimode 8-HR, Bruker) operated in peak force tapping mode using a SCANASYST-AIR probe (silicon tip on nitride lever, Bruker).

## Supporting Information

Supporting Information features Raman spectra of the MoSe_2_ flake, the absorption spectrum of the CuPc film, Raman spectra measured on CuPc/MoSe_2_ using a radial polarized beam, and a schematic diagram of the custom-built confocal optical microscope with a parabolic mirror.

File 1 Additional experimental data.
