# Supplementary material for: Revealing local structural properties of an atomically thin MoSe2 surface using optical microscopy
Source: Beilstein J Nanotechnol. 2022 Jul 1;13:572–81. doi: 10.3762/bjnano.13.49 (PMC9263549; doi:10.3762/bjnano.13.49)
Supplement: File 1 — Additional experimental data. [file Beilstein_J_Nanotechnol-13-572-s001.pdf]

BEILSTEIN JOURNAL OF NANOTECHNOLOGY

Supporting Information

for

Revealing local structural properties of an atomically thin $MoSe_2$ surface using optical microscopy

Lin Pan, Peng Miao, Anke Horneber, Alfred J. Meixner, Pierre-Michel Adam and Dai Zhang

Beilstein J. Nanotechnol. **2022,** *13,* 572–581. doi:10.3762/bjnano.13.49

Additional experimental data

License and Terms: This is a supporting information file under the terms of the Creative Commons Attribution License (https://creativecommons.org/licenses/by/4.0). Please note that the reuse, redistribution and reproduction in particular requires that the author(s) and source are credited and that individual graphics may be subject to special legal provisions.
The license is subject to the *Beilstein Journal of Nanotechnology* terms and conditions: (https://www.beilstein-journals.org/bjnano/terms)

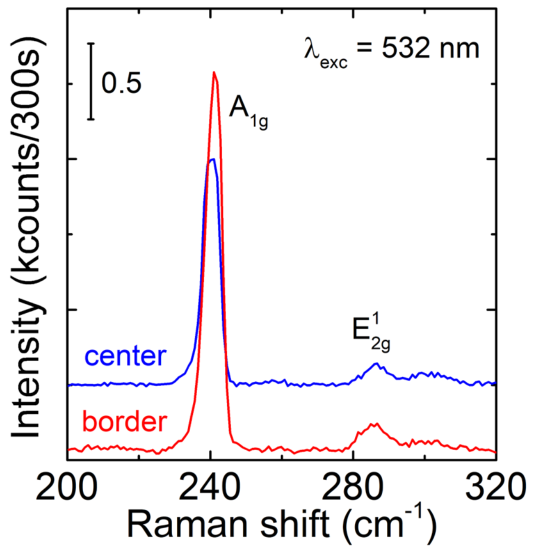

Figure S1: Raman spectra obtained from the border and center regions of a $MoSe_2$ flake with a 532 nm laser as the excitation source.

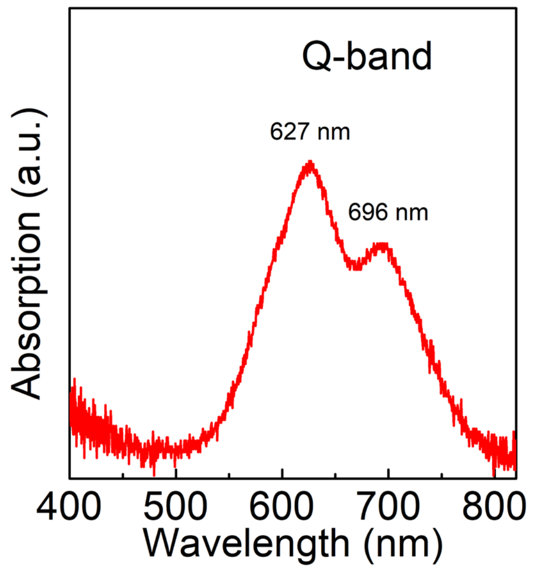

Figure S2: Absorption spectrum of a CuPc film with a thickness of 10 nm on a glass substrate. Two absorption peaks are located at 627 and 696 nm, which belong to the Q-band absorption of CuPc.

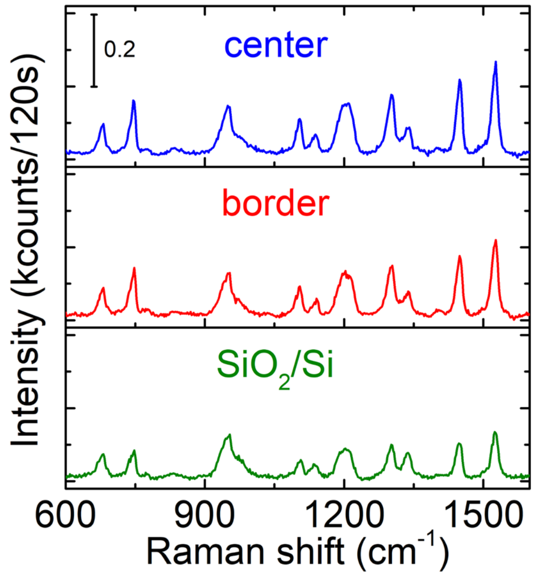

Figure S3: Raman spectra measured from CuPc/$MoSe_2$ using a radially polarized beam.

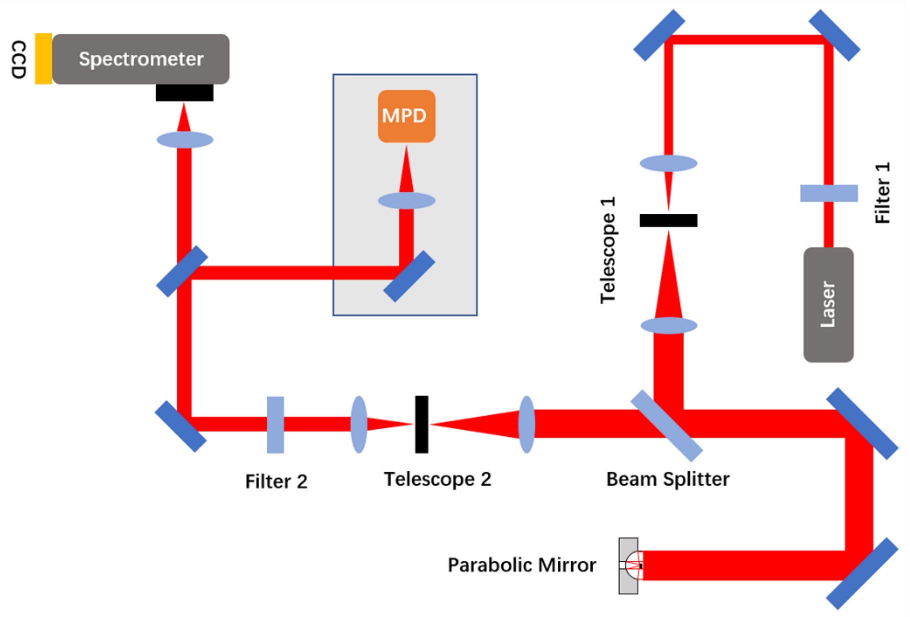

Figure S4: Schematic diagram of the custom-built confocal optical microscope with a parabolic mirror for light focusing and emission detection. Either a 636 nm (532 nm) CW laser or a 780 nm femtosecond pulsed laser are coupled to the microscope.
